# Supplementary material for: A supramolecular photosensitizer derived from an Arene-Ru(II) complex self-assembly for NIR activated photodynamic and photothermal therapy
Source: Nat Commun. 2022 Jun 2;13:3064. doi: 10.1038/s41467-022-30721-w (PMC9163081; doi:10.1038/s41467-022-30721-w)
Supplement: Supplementary file 1 — Supplementary Information [file 41467_2022_30721_MOESM1_ESM.pdf]

A Supramolecular Photosensitizer Derived from an Arene-Ru(II) Complex Self-assembly for NIR Activated Photodynamic and Photothermal Therapy

Gang Xu [1], Chengwei Li [1], Chen Chi [2], Luyan Wu [2], Yanyan Sun [3], Jian Zhao [1], Xing-Hua Xia [2]*, Shaohua Gou [1]**

1, Jiangsu Province Hi-Tech Key Laboratory for Biomedical Research and Pharmaceutical Research Center, School of Chemistry and Chemical Engineering, Southeast University, Nanjing 211189, China

2, State Key Lab of Analytical Chemistry for Life Science, School of Chemistry and Chemical Engineering, Nanjing University, Nanjing 210023, China.

3, School of Chemistry and Life Sciences, Suzhou University of Science and Technology, Suzhou 215009, China

E-mail: zhaojianzhaokuan@163.com (J. Zhao), xhxia@nju.edu.cn (X.-H. Xia), sgou@seu.edu.cn (S. Gou)

Table of contents

Supplementary Figure 1. Synthetic route of RuDA and RuET.

Supplementary Figure 2. ^{1}H NMR spectrum of RuDA.

Supplementary Figure 3. ^{13}C NMR spectrum of RuDA.

Supplementary Figure 4. ESI-MS diagram of RuDA.

Supplementary Figure 5. Electron density difference maps (EDDMs) of the lowest-lying singlet transitions for RuDA.

Supplementary Figure 6. Normalized emission of RuDA in different solutions.

Supplementary Figure 7. In-liquid AFM image of RuDA in (A) CH_3OH, and (B) a CH_3OH/H_2O mixture with water fraction of 90%. A representative image of three independent tests from each group is shown.

Supplementary Figure 8. UV-vis absorption spectra of (A) RuDA, and (B) ICG in aqueous solution under 808 nm laser irradiation.

Supplementary Figure 9. UV-vis absorption spectra of RuDA-NPs in PBS (A) pH=5.4, (B) pH=7.4, (C) pH=9.0, (D) FBS, and (E) DMEM at different time points.

Supplementary Figure 10. HPLC chromatograms of RuDA (50 μM) in methanol and water solution (50/50, v/v) after (A) 0 h, (B) 1 h, and (C) 4 h incubation.

Supplementary Figure 11. GPC profiles of (A) RuDA-NPs (50 μM) in PBS buffer (pH = 7.4) after different incubation times (0h, 1h, 8h). (B) Water.

Supplementary Figure 12. TEM images of RuDA-NPs after incubation in PBS (pH = 7.4) for (A) 0 h, and (B) 24 h. A representative image of three independent tests from each group is shown.

Supplementary Figure 13. Time-dependent fluorescence spectra of ABDA (50 μM) for RuDA (20 μM) in DMF/H_2O mixtures with water fractions: (A) 0%, (B) 20%, (C) 40%, (D) 60%, (E) 80%, F) 95% under 808 nm laser irradiation.

Supplementary Figure 14. EPR signals of TEMPO and DMPO-OH· adducts for (A) $^1O_2$ and (B) ·OH characterization upon NIR (808 nm, 0.5 W cm^{-2}) irradiation of the mixture of RuDA (50 μM) and TEMP (20 mM) or DMPO (20 mM) at 0, 2, and 4 min, respectively.

Supplementary Figure 15. Synthetic route of **L2**.

Supplementary Figure 16. ^{1}H NMR spectrum of **L2**.

Supplementary Figure 17. ^{13}C NMR spectrum of **L2**.

Supplementary Figure 18. ESI-MS diagram of **L2**.

Supplementary Figure 19. ^{1}H NMR spectrum of RuET.

Supplementary Figure 20. ^{13}C NMR spectrum of RuET.

Supplementary Figure 21. ESI-MS diagram of RuET.

Supplementary Figure 22. In-liquid AFM image of RuET in a CH_3OH/H_2O mixture with water fraction of 90%. A representative image of three independent tests from each group is shown.

Supplementary Figure 23. Time-dependent fluorescence spectra of ABDA (50 μM) for RuET (20 μM) in DMF/H_2O mixtures with 95% water fractions under 808 nm laser (0.5 W cm^{-2}) irradiation.

Supplementary Figure 24. UV-vis absorption spectra of RuDA in DMF and RuDA-NPs in water.

Supplementary Figure 25. Flow cytometry and quantitative analysis of ROS levels. (A) Flow cytometry analysis of ROS levels in MDA-MB-231 cells after different treatment conditions; (B) Quantitative analysis of the mean fluorescent intensity (MFI) of DCF. The results are mean ± SD (n = 3). (***) $p < 0.001$ compared with the value of the control (PBS + irradiation) group (unpaired, two-sided t tests).

Supplementary Figure 26. Ruthenium uptake (ng/mg protein) in the different cellular compartments of MDA-MB-231 cells (treated with RuDA or RuDA-NPs at 50 μM for 12 h) quantified by ICP-MS. The data represent the mean ± SD (n = 3).

Supplementary Figure 27. JC-1 and MitoSOX Red staining of MDA-MB-231 cells treated with RuDA-NPs (50 μM) or RuDA (50 μM) upon 808 nm laser (0.5 W cm^{-2}) irradiation for 10 min. Scale bars: 30 μm. A representative image of three biological replicates from each group is shown.

Supplementary Figure 28. An example of gating strategy on MDA-MB-23 cells for flow cytometric study in **Figure 6E.**

Supplementary Figure 29. Quantification of Nrf2, Hsp70, and HO-1 expressions of MDA-MB-231 cells treated with PBS, RuDA-NPs (50 μM) for 12 h with or without 808 nm laser irradiation (0.5 W cm^{-2}, 10 min); the intensities of Nrf2, Hsp70, and HO-1 were normalized to that of β-actin using Gel-Pro 32 software. The data represent the mean ± SD (n = 2).

Supplementary Figure 30. *In vivo* PA images of tumor sites under excitation at 808 nm after (A) intratumoral injection of RuDA-NPs (5 μmol kg^{-1} and 20 μmol kg^{-1}). (B) intravenous injection of RuDA (10 μmol kg^{-1}) at different time points.

Supplementary Figure 31. Ru excreted out of the mice body *via* urine and feces after intravenous administration of RuDA (10 μmol kg^{-1}) at different time intervals. The data represent the mean ± SD (n =3).

Supplementary Figure 32. Temperature elevation of mice after 10 min irradiation (808 nm, 0.5 W cm^{-2}), which is the quantitative data of **Figure 8A**. Error bars, mean ± SD (n = 4).

Supplementary Figure 33. Tumor images from different groups of mice after 15 days' therapy.

Supplementary Table 1. Distribution of holes, electrons, and overlaps of the low-energy singlet excited states for RuDA in the monomeric form.

Supplementary Table 2. Distribution of holes, electrons, and overlaps of the low-energy singlet excited states for RuDA in the dimeric form.

Supplementary Table 3. Contribution ratio (η) of RuDA in the dimeric form for the electron transition from one RuDA molecule to the other one for the low-energy singlet excited states.

Supplementary Table 4. Singlet and triplet excited states transition configurations of monomeric RuDA revealed by TD-DFT calculations.

Supplementary Table 5. Singlet and triplet excited states transition configurations of dimeric RuDA revealed by TD-DFT calculations.

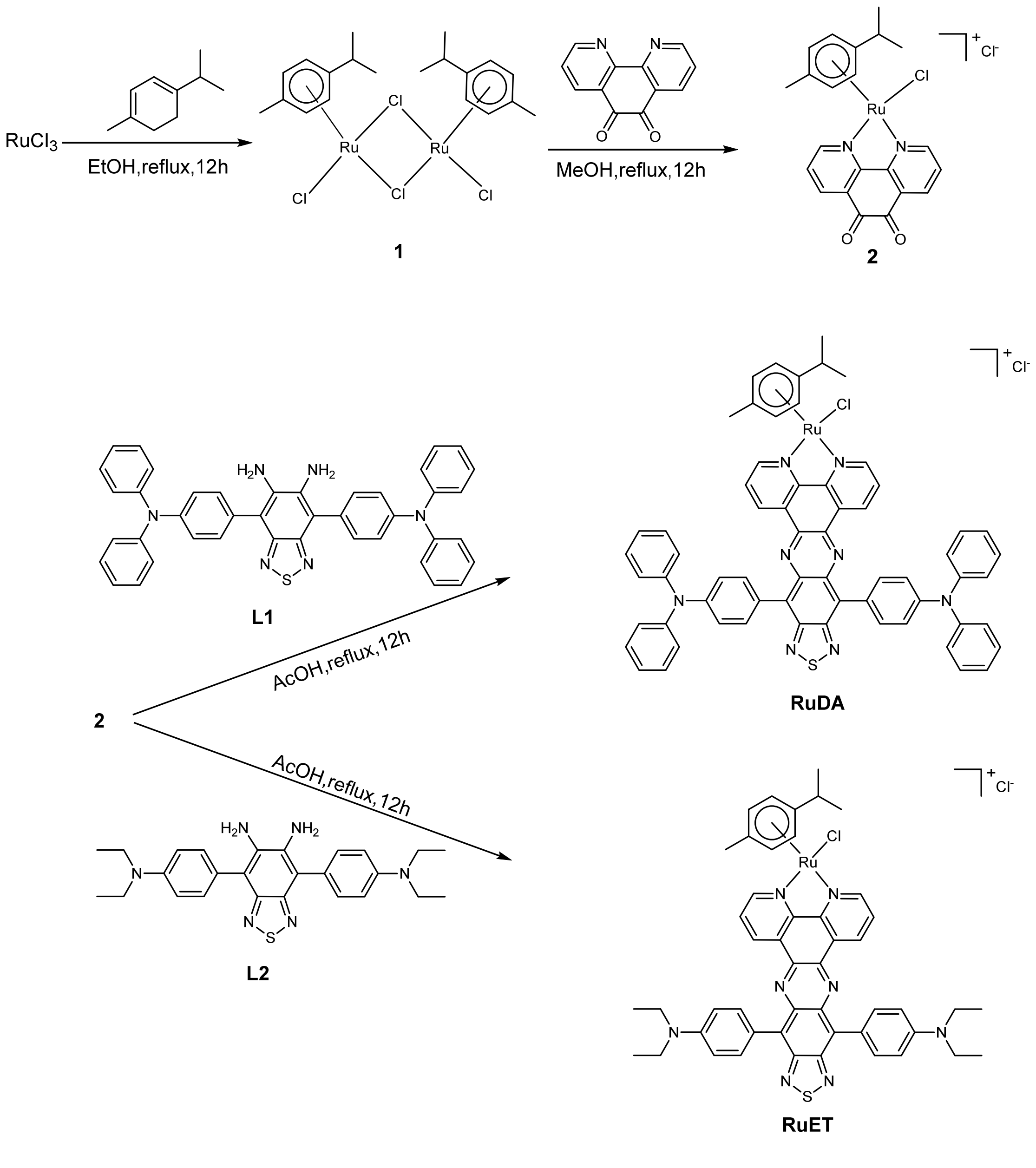

Supplementary Figure 1. Synthetic route of RuDA and RuET.

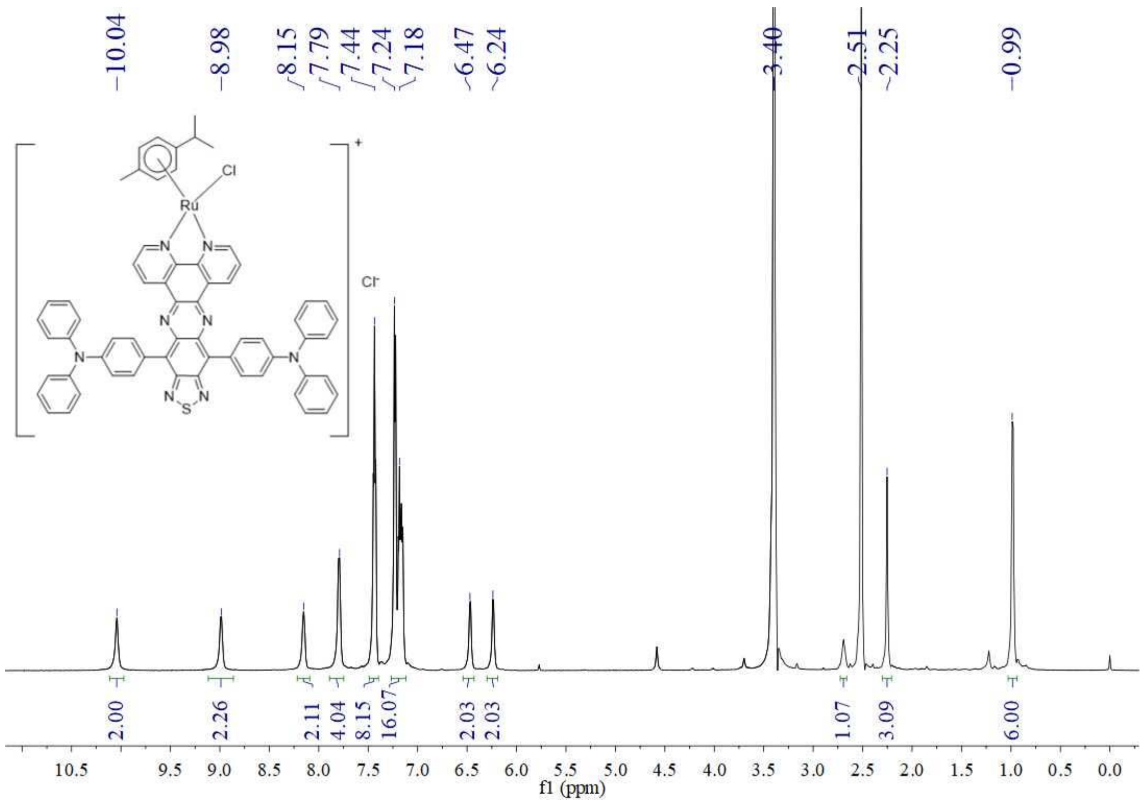

Supplementary Figure 2. ^{1}H NMR spectrum of RuDA.

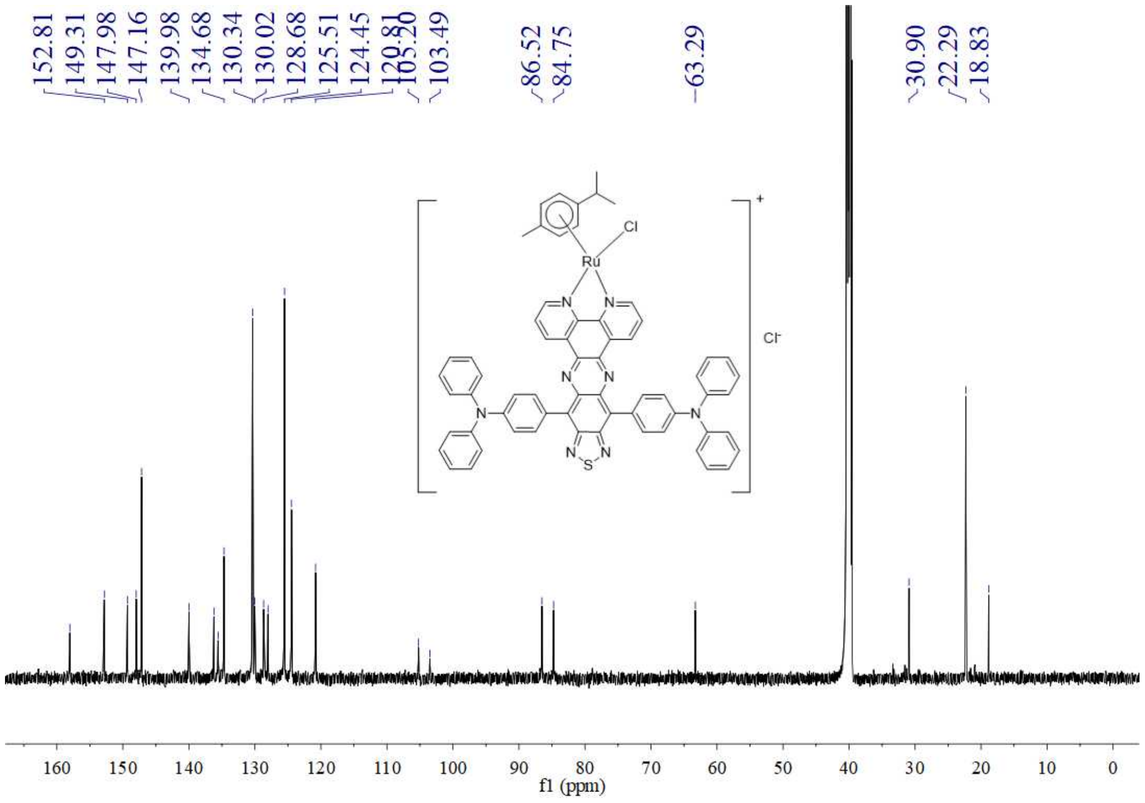

Supplementary Figure 3. ^{13}C NMR spectrum of RuDA.

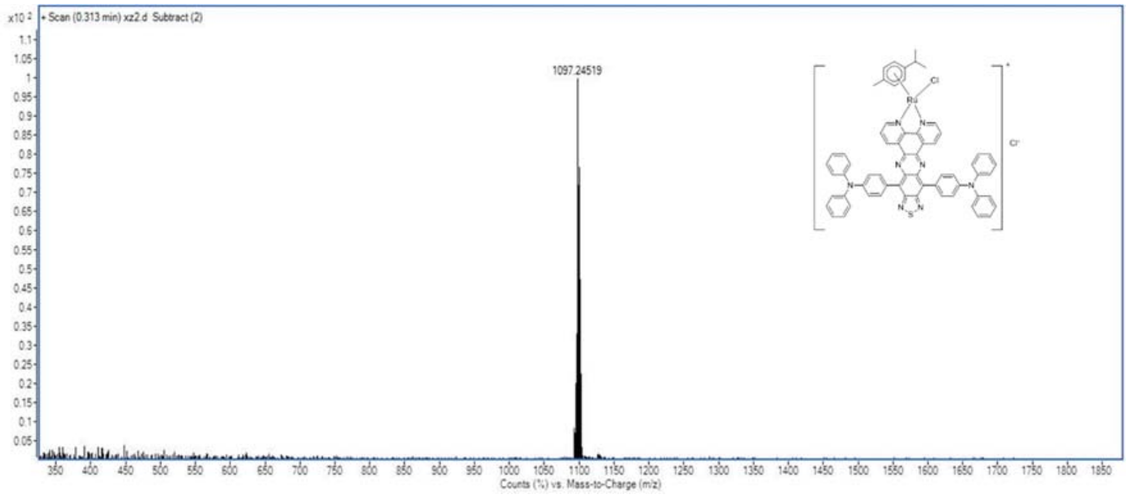

Supplementary Figure 4. ESI-MS diagram of RuDA.

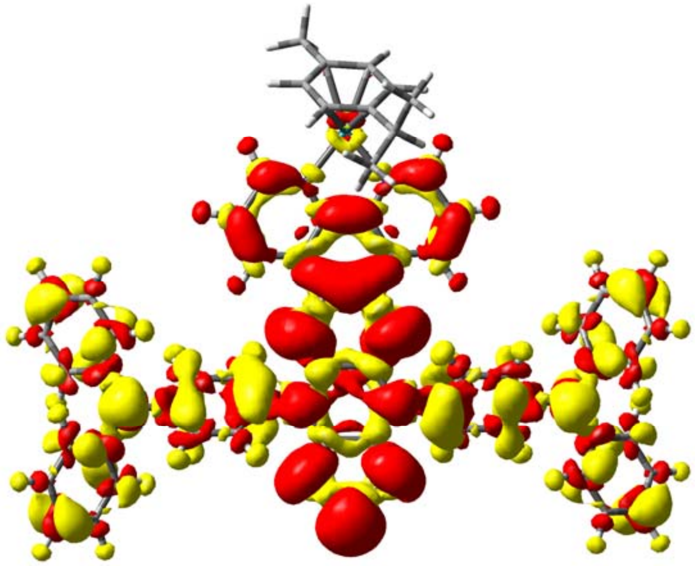

Supplementary Figure 5. Electron density difference maps (EDDMs) of the lowest-lying singlet transitions for RuDA (yellow indicates a decrease in charge density, while orange indicates an increase).

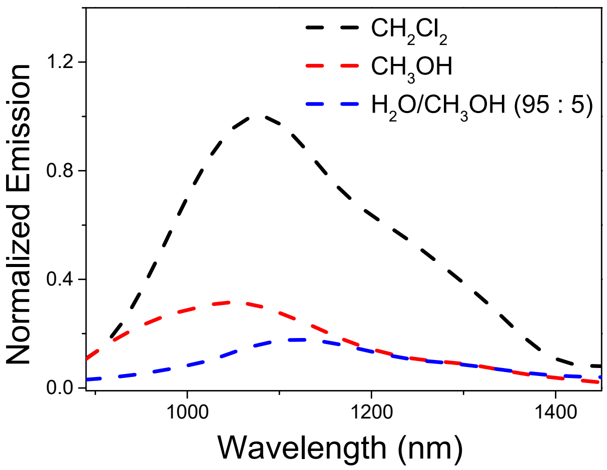

Supplementary Figure 6. Normalized emission of RuDA in different solutions.

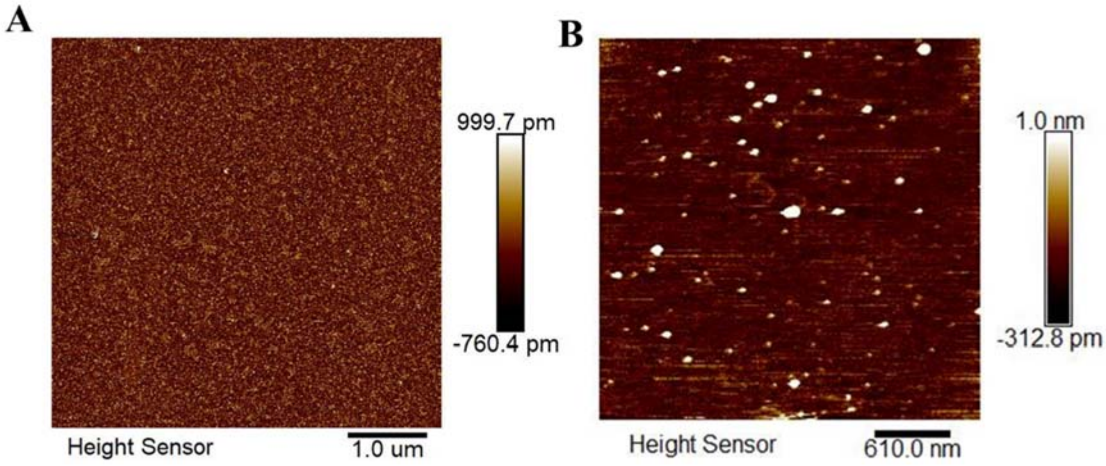

Supplementary Figure 7. In-liquid AFM image of RuDA in (A) CH_3OH, and (B) a CH_3OH/H_2O mixture with water fraction of 90%. A representative image of three independent tests from each group is shown.

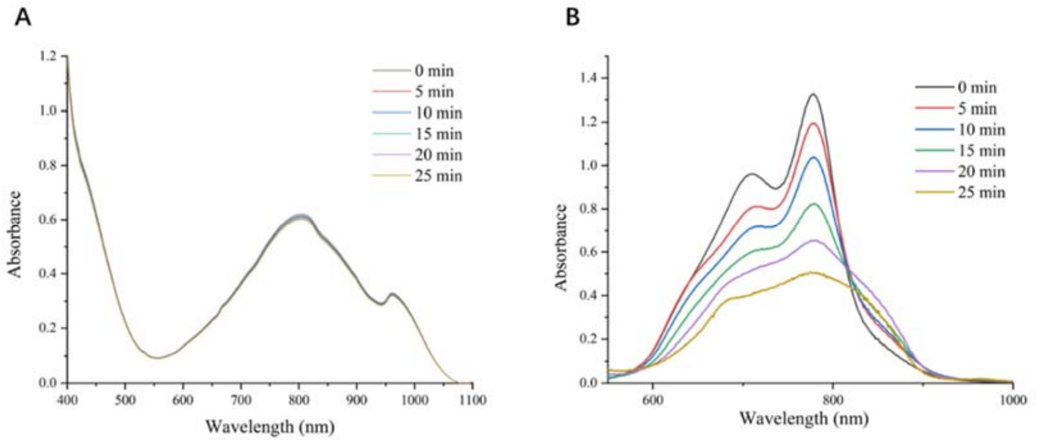

Supplementary Figure 8. UV-vis absorption spectra of (A) RuDA, and (B) ICG in aqueous solution under 808 nm laser irradiation.

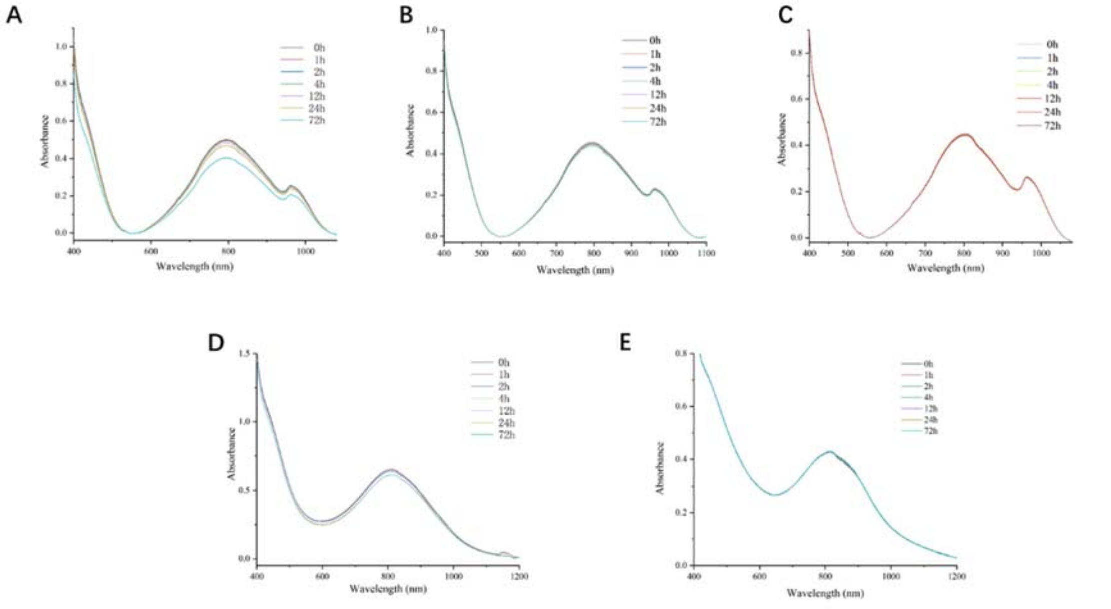

Supplementary Figure 9. UV-vis absorption spectra of RuDA-NPs in PBS (A) pH=5.4, (B) pH=7.4, (C) pH=9.0, (D) FBS, and (E) DMEM at various time points.

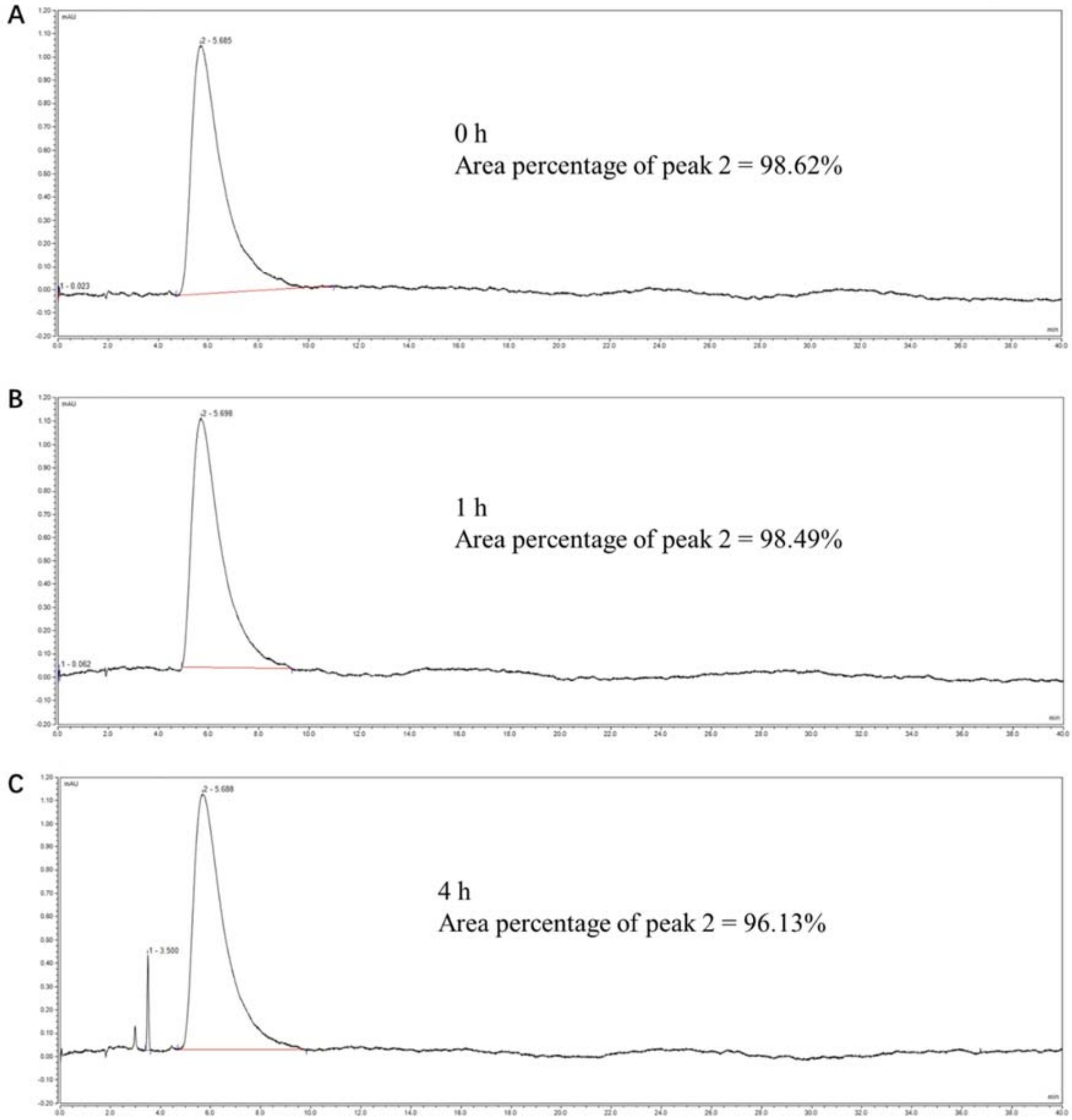

Supplementary Figure 10. HPLC chromatograms of RuDA (50 μM) in a solution of methanol and water (50/50, v/v) after (A) 0 h, (B) 1 h, and (C) 4 h incubation.

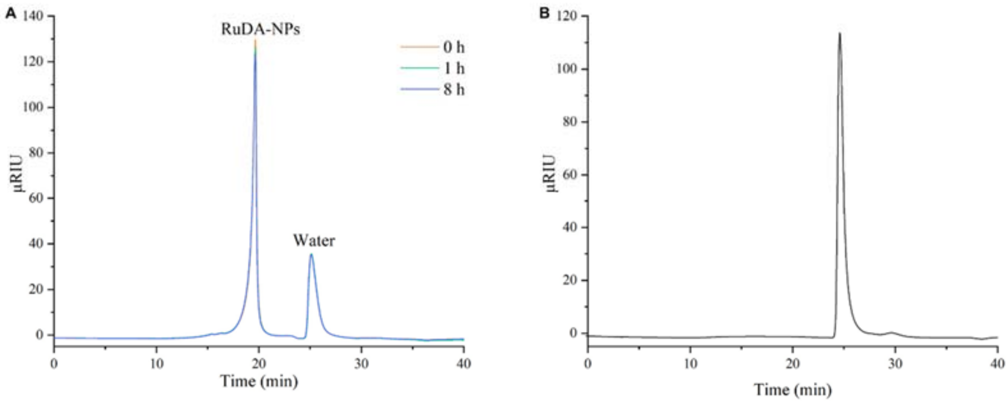

Supplementary Figure 11. GPC profiles of (A) RuDA-NPs (50 μM) in PBS buffer (pH = 7.4) after different incubation times (0h, 1h, 8h). (B) Water.

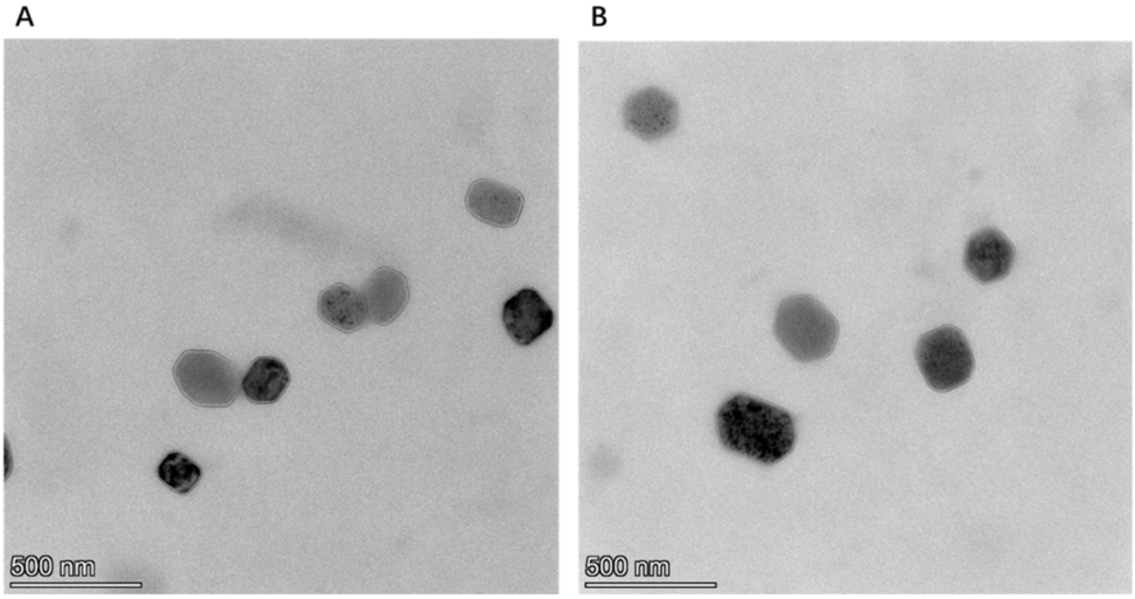

Supplementary Figure 12. TEM images of RuDA-NPs after incubation in PBS (pH = 7.4) for (A) 0 h, and (B) 24 h. A representative image of three independent tests from each group is shown.

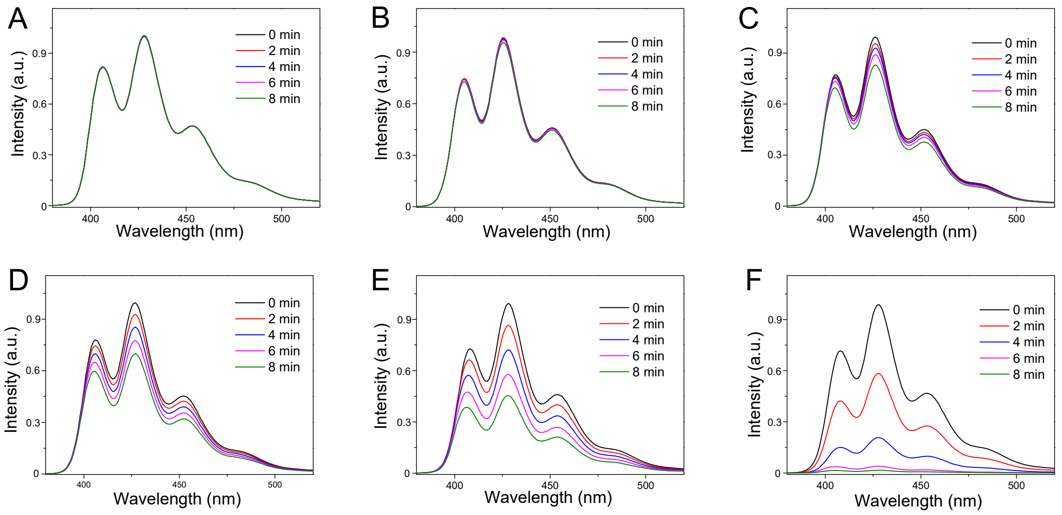

Supplementary Figure S13. Time-dependent fluorescence spectra of ABDA (50 μM) for RuDA (20 μM) in DMF/H_2O mixtures with water fractions: (A) 0%, (B) 20%, (C) 40%, (D) 60%, (E) 80%, and (F) 95% under 808 nm laser irradiation.

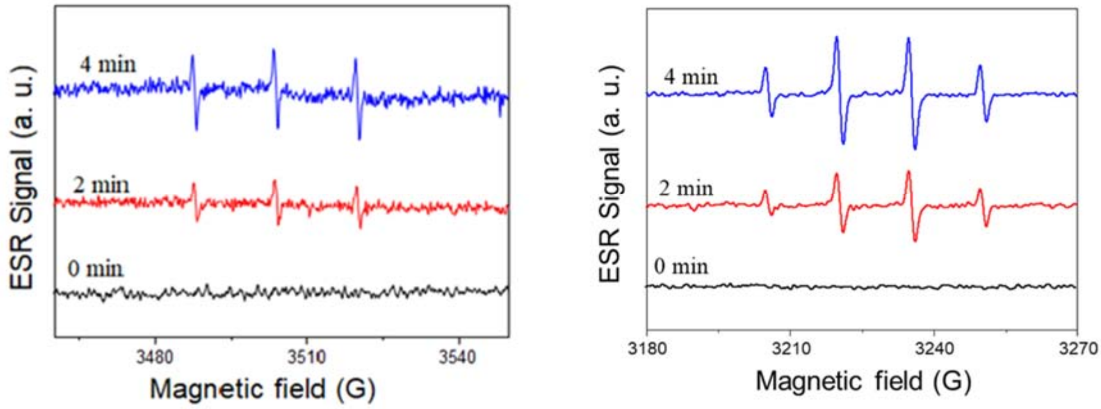

Supplementary Figure 14. EPR signals of TEMPO and DMPO-OH· adducts for (A) $^1O_2$ and (B) ·OH characterization upon NIR (808 nm, 0.5 W cm^{-2}) irradiation of the mixture of RuDA (50 μM) and TEMP (20 mM) or DMPO (20 mM) at 0, 2, and 4 min, respectively.

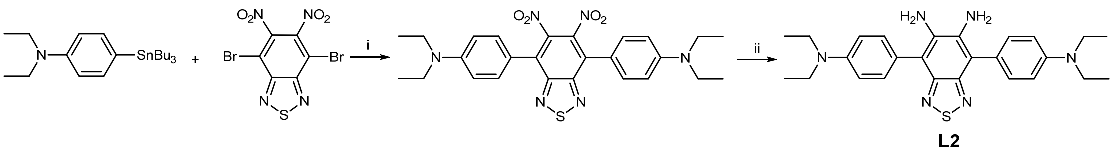

Supplementary Figure 15. Synthetic route of **L2**. Reagent and conditions: i) Pd(PPh3)4, toluene, 100 ºC, 24 h; ii) Fe, AcOH, 80 ºC, 3 h.

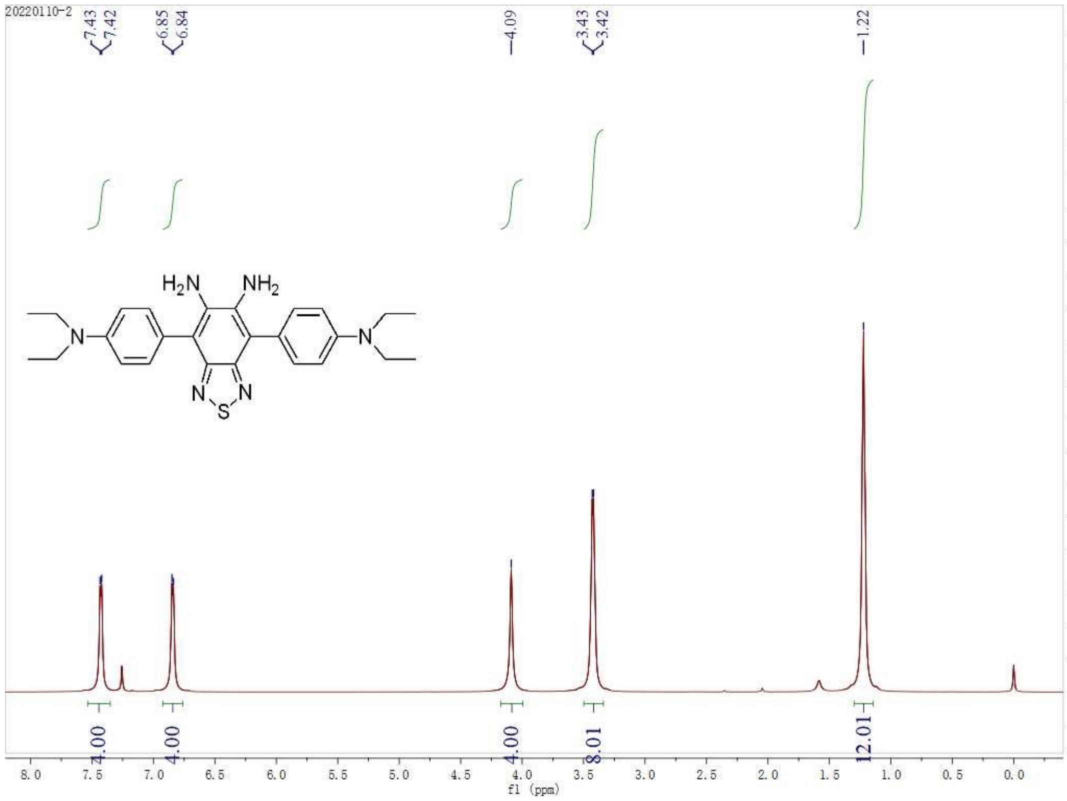

Supplementary Figure 16. ^{1}H NMR spectrum of **L2**.

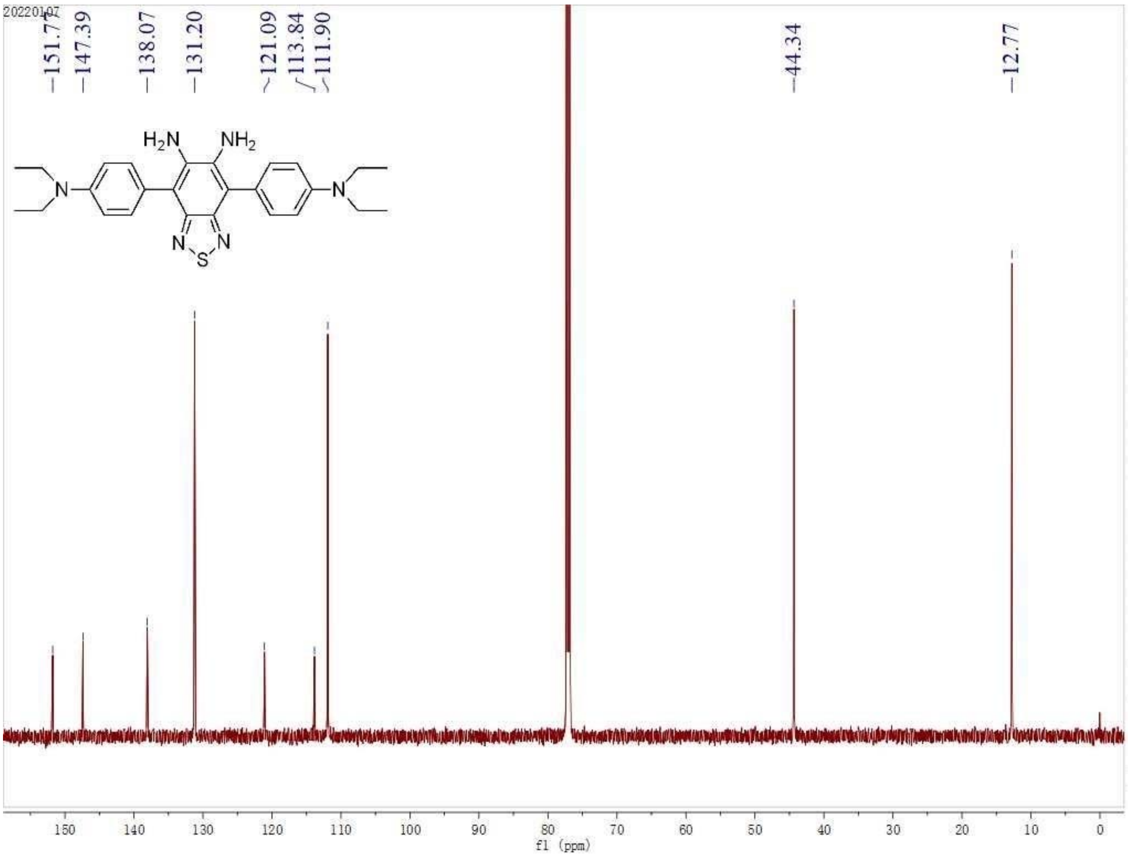

Supplementary Figure 17. ^{13}C NMR spectrum of **L2**.

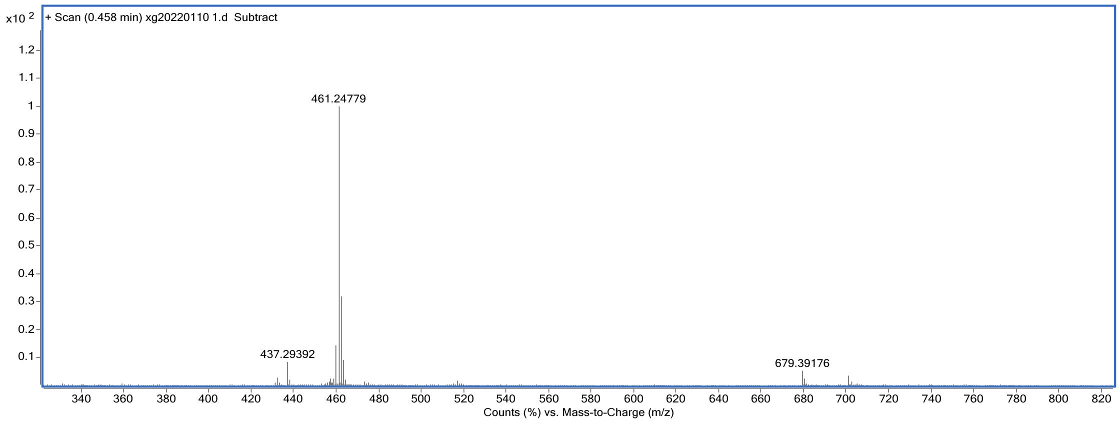

Supplementary Figure 18. ESI-MS diagram of **L2**.

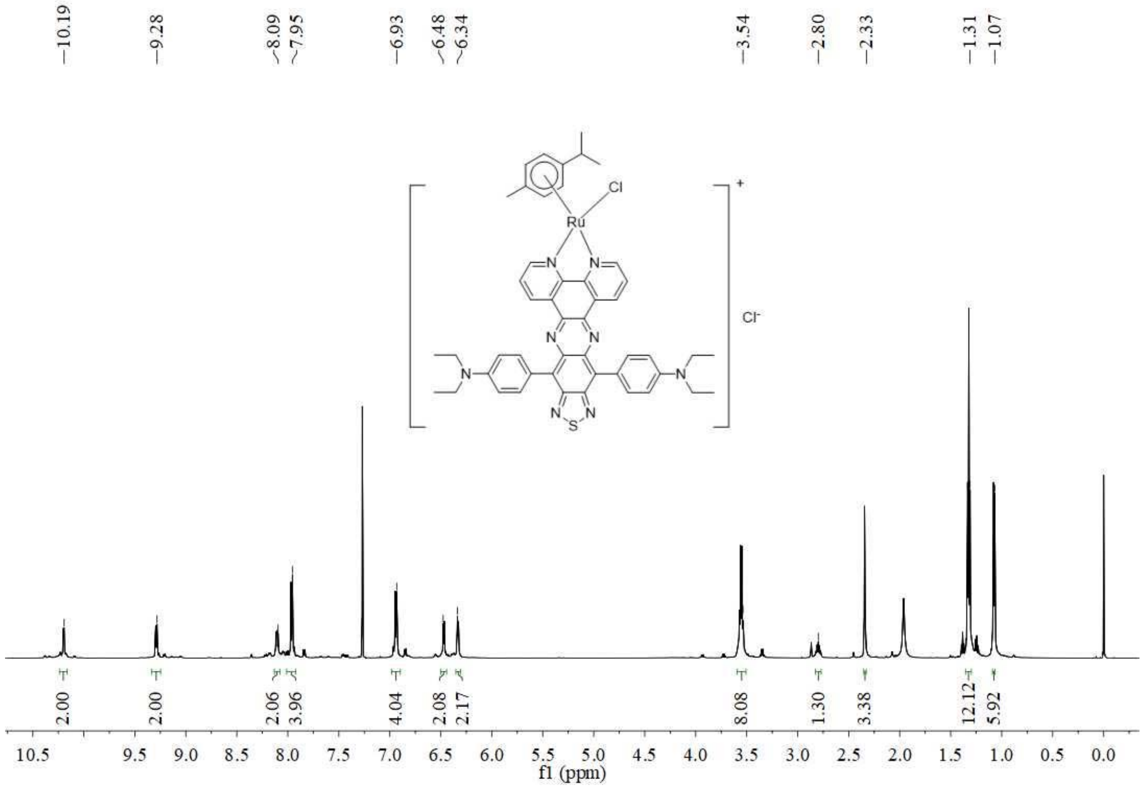

Supplementary Figure 19.^{1}H NMR spectrum of RuET.

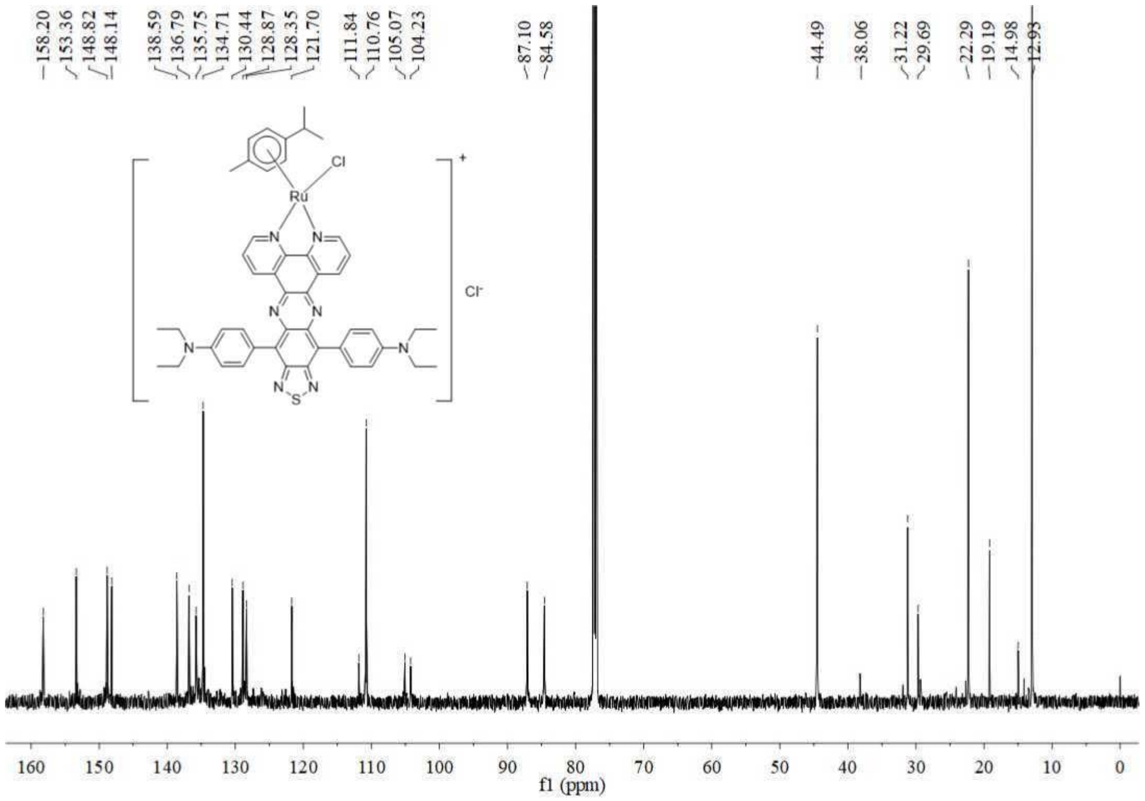

Supplementary Figure 20. ^{13}C NMR spectrum of RuET.

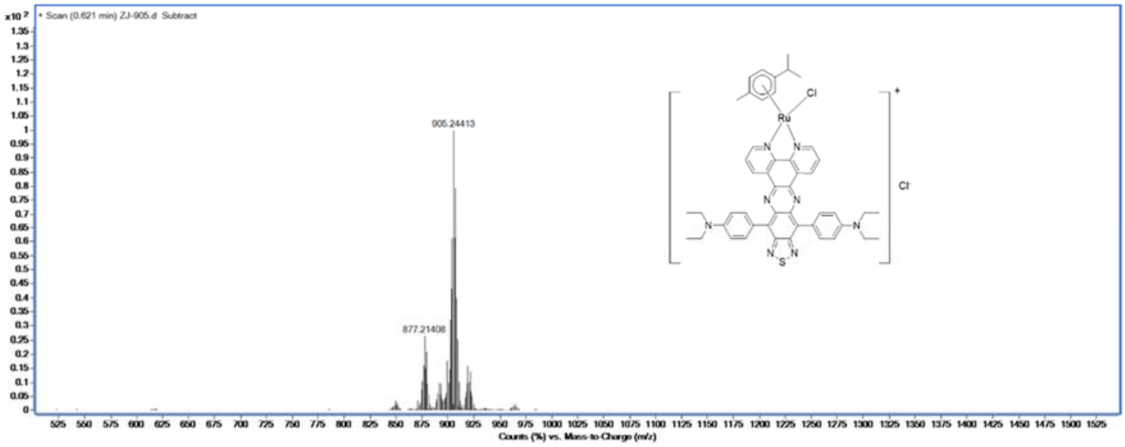

Supplementary Figure 21. ESI-MS diagram of RuET.

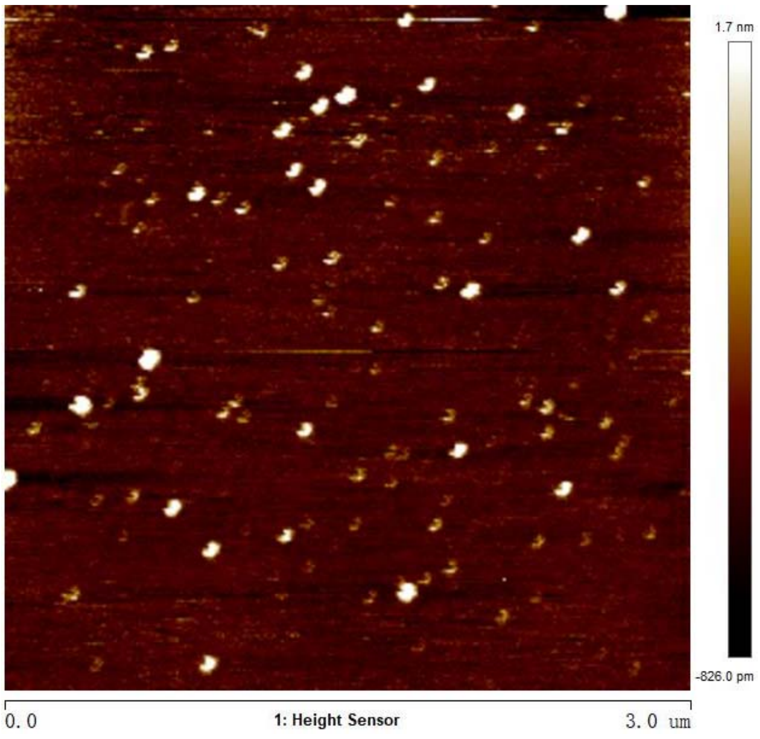

Supplementary Figure 22. In-liquid AFM image of RuET in a CH_3OH/H_2O mixture with water fraction of 90%. A representative image of three independent tests from each group is shown.

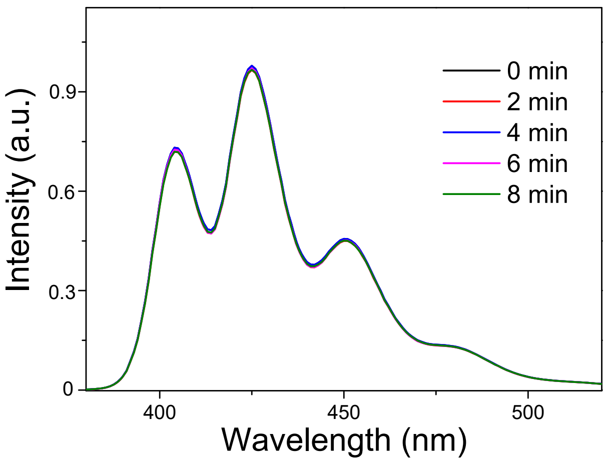

Supplementary Figure 23. Time-dependent fluorescence spectra of ABDA (50 μM) for RuET (20 μM) in DMF/H_2O mixtures with 95% water fractions under 808 nm laser irradiation.

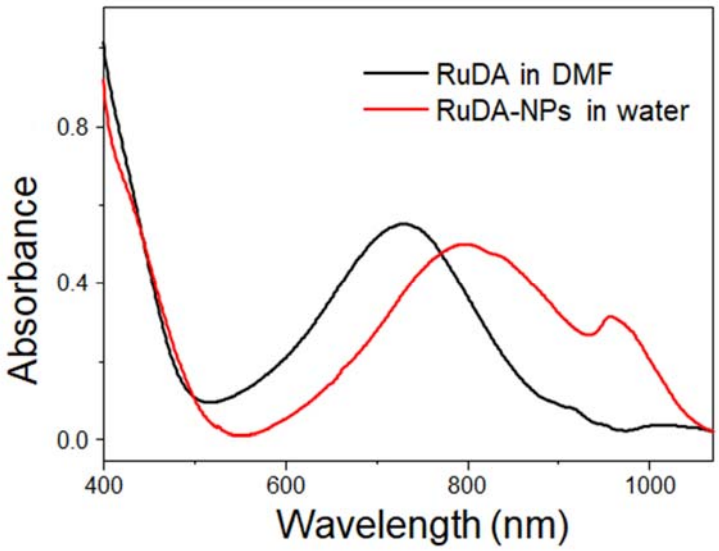

Supplementary Figure 24. UV-vis absorption spectra of RuDA in DMF and RuDA-NPs in water.

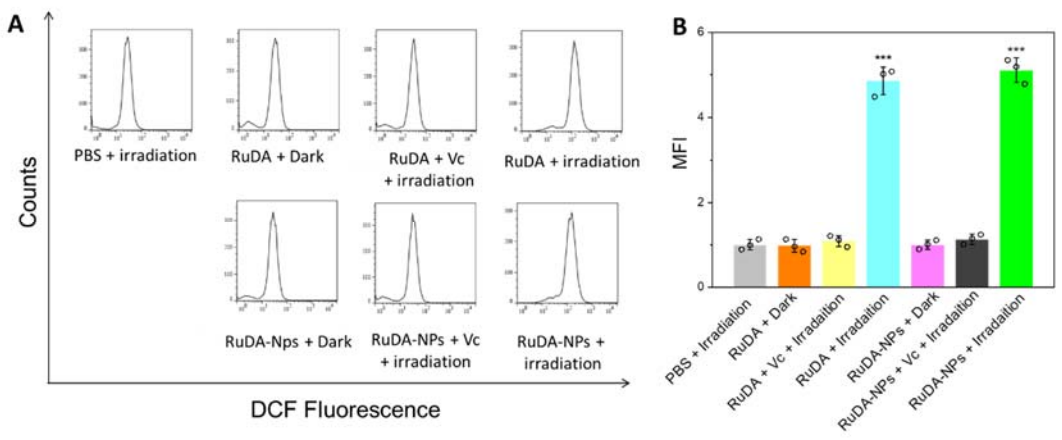

Supplementary Figure 25. Flow cytometry and quantitative analysis of ROS levels. (A) Flow cytometry analysis of ROS levels in MDA-MB-231 cells after different treatment conditions; (B) Quantitative analysis of the mean fluorescent intensity (MFI) of DCF. The results are mean ± SD (n = 3). P < 0.001 (***) compared with the value of the control (PBS + irradiation) group (unpaired, two-sided t tests).

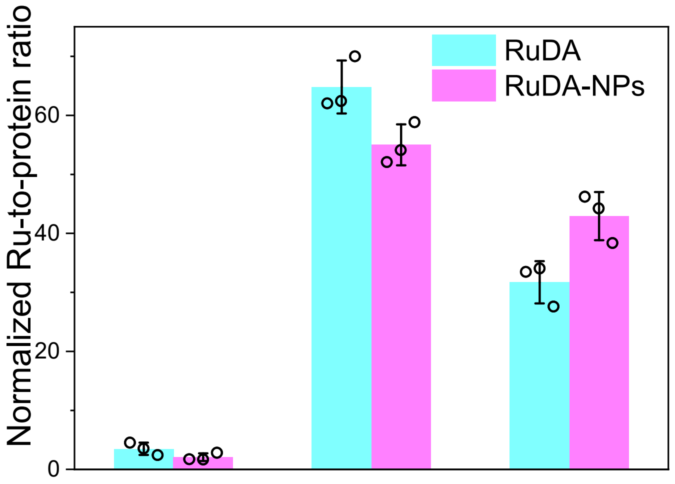

Supplementary Figure 26. Ruthenium uptake (ng/mg protein) in the different cellular compartments of MDA-MB-231 cells treated with RuDA or RuDA-NPs at 50 µM for 12 h quantified by ICP-MS. The data represent the mean ± SD (n = 3).

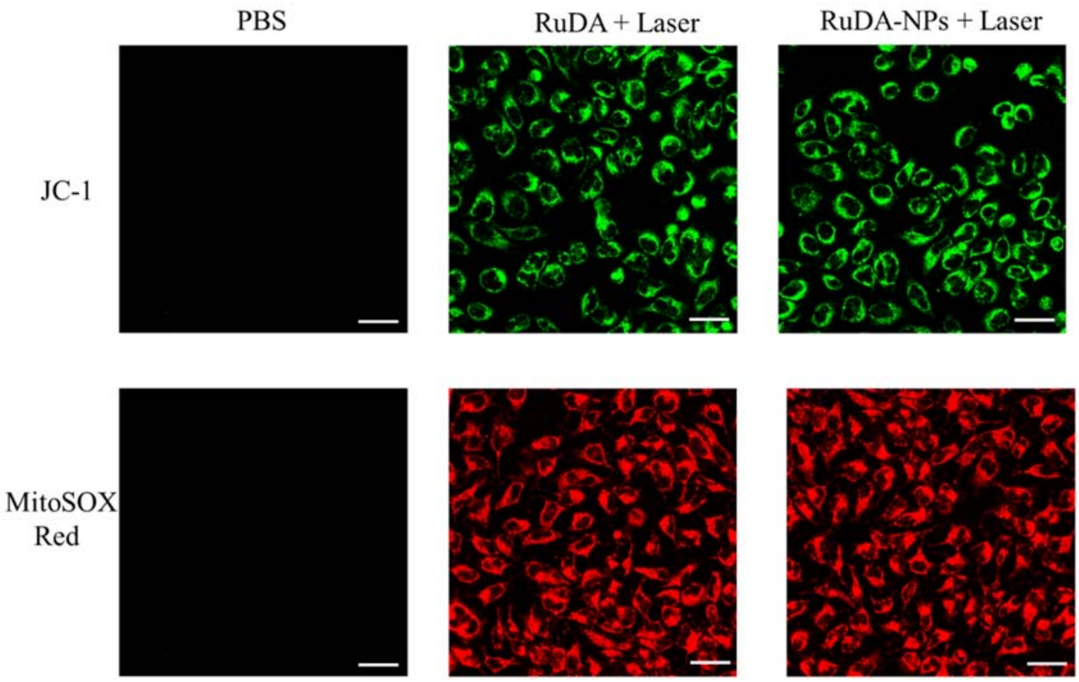

Supplementary Figure 27. JC-1 and MitoSOX Red staining of MDA-MB-231 cells treated with RuDA-NPs (50 μM) or RuDA (50 μM) upon 808 nm laser (0.5 W cm^{-2}) irradiation for 10 min. Scale bars: 30 μm. A representative image of three biological replicates from each group is shown.

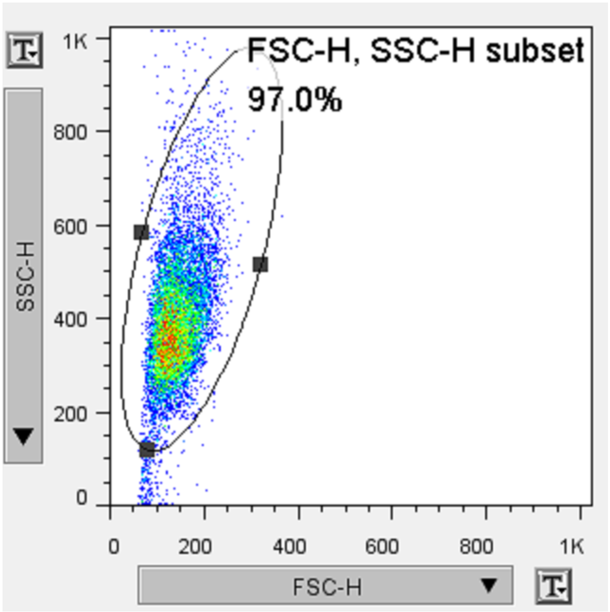

Supplementary Figure 28. An example of gating strategy on MDA-MB-231cells for flow cytometric study in **Figure 6E.**

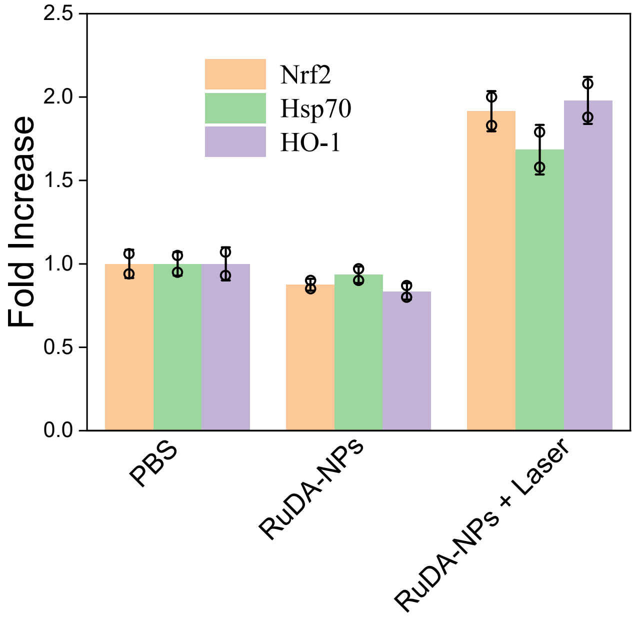

Supplementary Figure 29. Quantification of Nrf2, Hsp70, and HO-1 expressions of MDA-MB-231 cells treated with PBS, RuDA-NPs (50 μM) for 12 h with or without 808 nm laser irradiation (0.5 W cm^{-2}, 10 min); the intensities of Nrf2, Hsp70, and HO-1 were normalized to that of β-actin using Gel-Pro 32 software. The data represent the mean ± SD (n = 2).

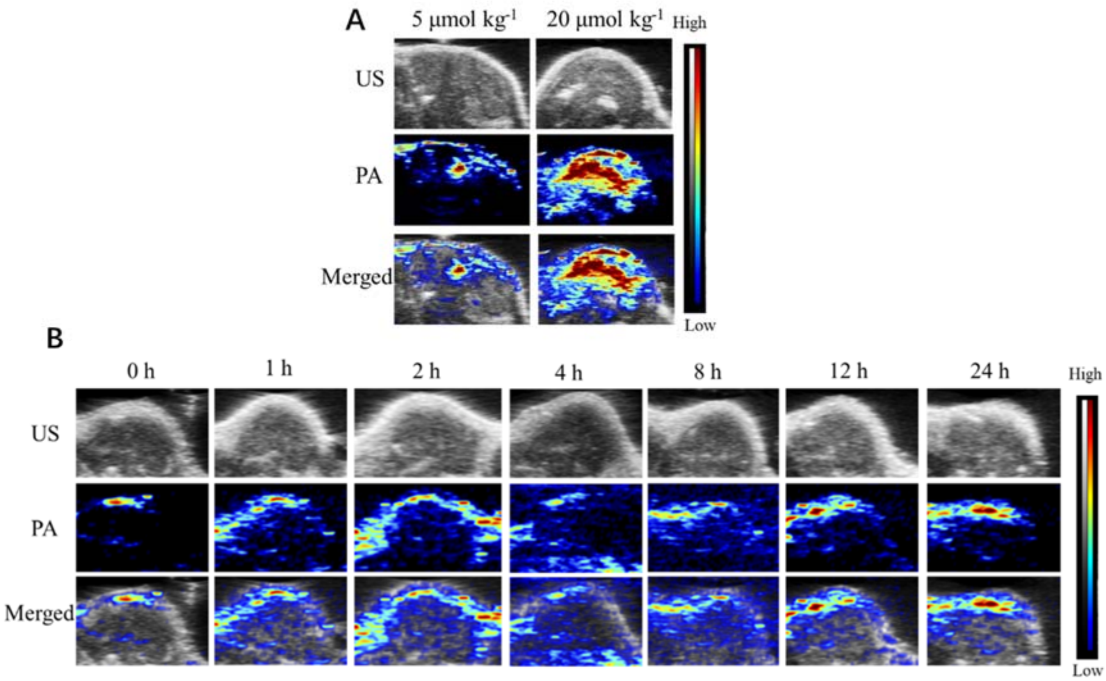

Supplementary Figure 30. *In vivo* PA images of tumor sites under excitation at 808 nm after (A) intratumoral injection of RuDA-NPs (5 μmol kg^{-1} and 20 μmol kg^{-1}). (B) intravenous injection of RuDA (10 μmol kg^{-1}) at different time points.

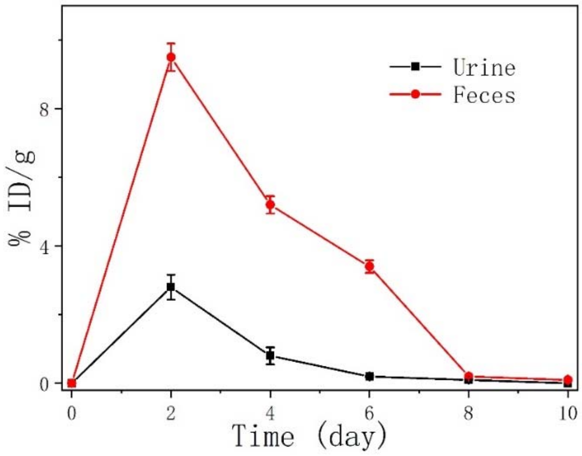

Supplementary Figure 31. Ru excreted out of the mice body *via* urine and feces after intravenous administration of RuDA (10 μmol kg^{-1}) at different time intervals. The data represent the mean ± SD (n =3).

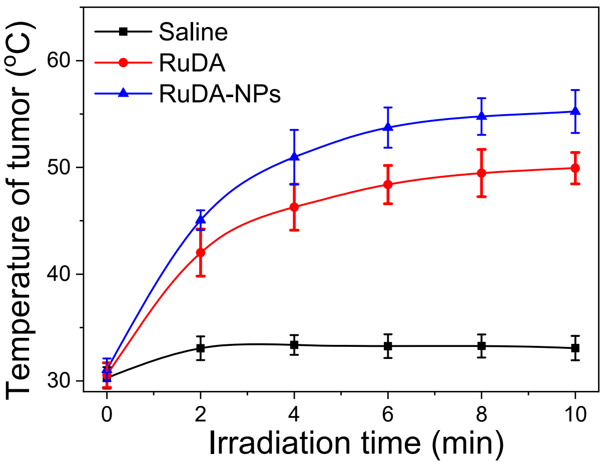

Supplementary Figure 32. Temperature elevation of mice after 10 min irradiation (808 nm, 0.5 W cm^{-2}), which is the quantitative data of **Figure 8A**. Error bars, mean ± SD (n = 4).

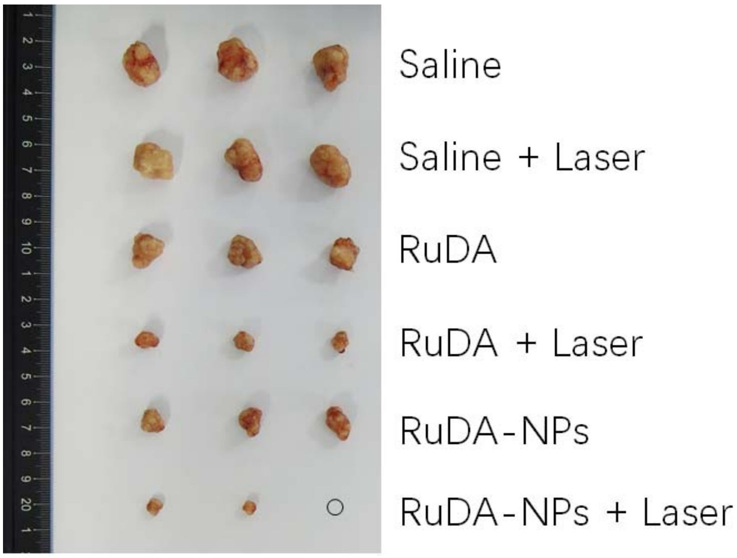

Supplementary Figure 33. Tumor images from different groups of mice after 15 days' therapy.

Supplementary Table 1. Distribution of holes, electrons, and overlaps of the low-energy singlet excited states for RuDA in the monomeric form.

Excited state and properties	Holes	Electrons	Overlap
S_1<br>1.30 eV<br>f = 0.4228	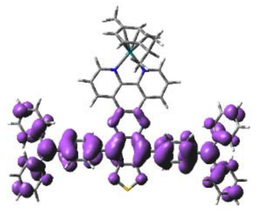	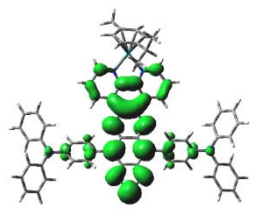	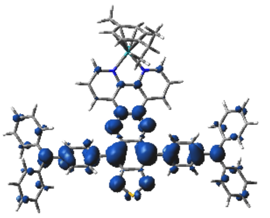
S_2<br>1.64 eV<br>f = 0.0212	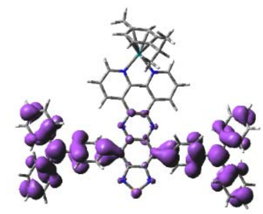	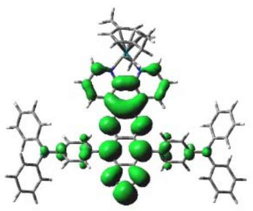	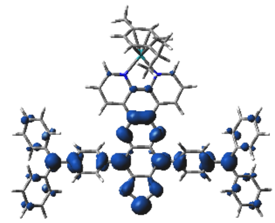
S_3<br>2.28 eV<br>f = 0.0012	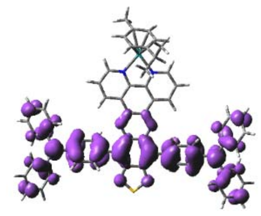	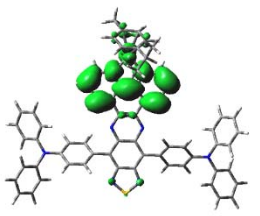	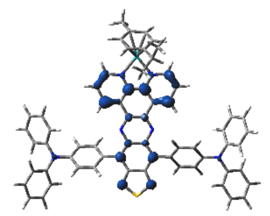
S_4<br>2.39 eV<br>f = 0.0001	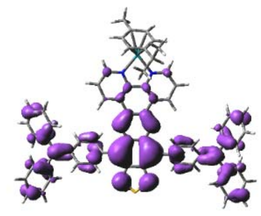	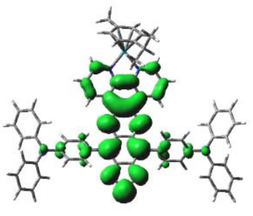	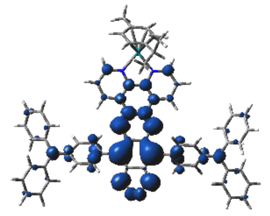
S_5<br>2.61 eV<br>f = 0.0128	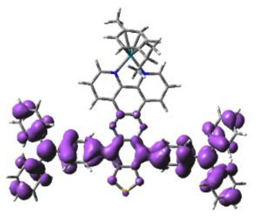	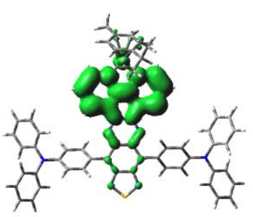	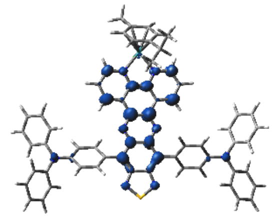
S_6<br>2.67 eV<br>f = 0.0449	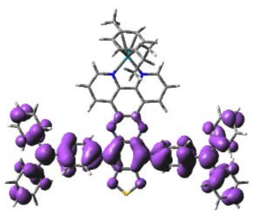	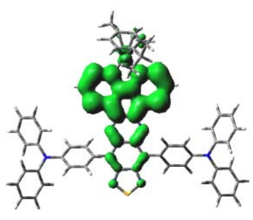	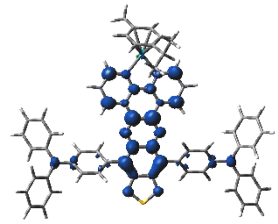

Supplementary Table 2. Distribution of holes, electrons, and overlaps of the low-energy singlet excited states for RuDA in the dimeric form.

Excited state and properties	Holes	Electrons	Overlap
S_1<br>1.27 eV<br>$f = 0.0280$	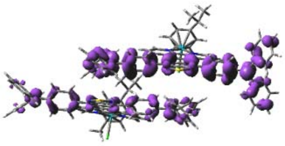	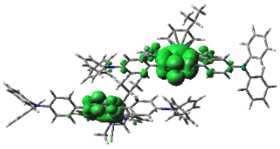	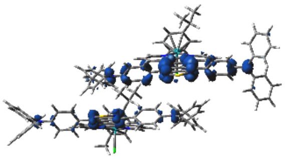
S_2<br>1.28 eV<br>$f = 0.6370$	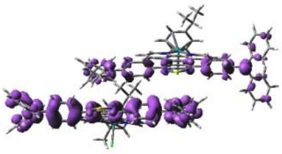	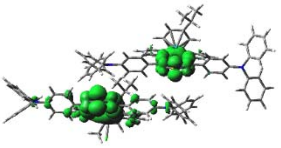	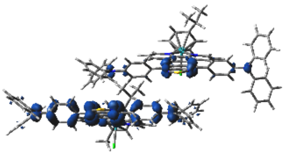
S_3<br>1.29 eV<br>$f = 0.0232$	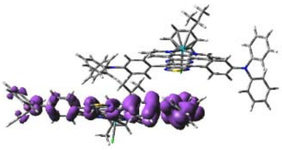	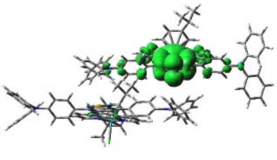	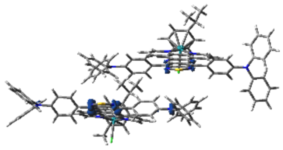
S_4<br>1.31 eV<br>$f = 0.0299$	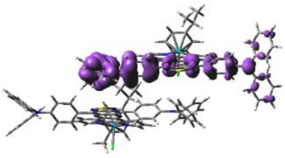	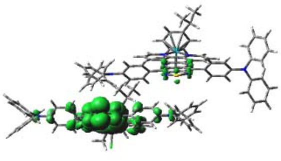	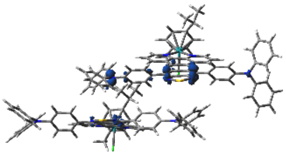
S_5<br>1.59 eV<br>$f = 0.0316$	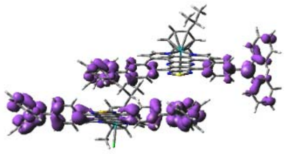	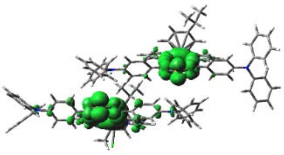	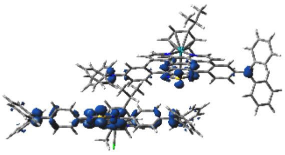
S_6<br>1.61 eV<br>$f = 0.0040$	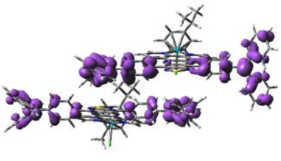	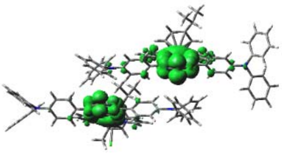	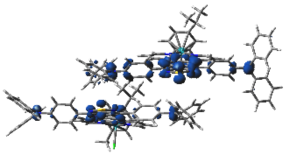

S$_7$
1.69 eV
f = 0.0000

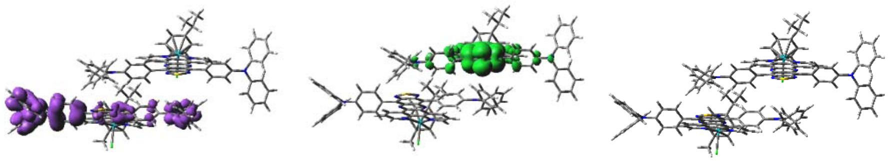

S$_8$
1.70 eV
f = 0.0001

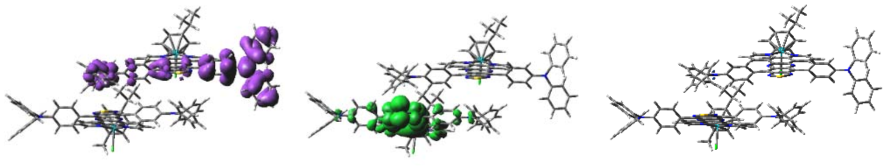

S$_9$
2.27 eV
f = 0.0009

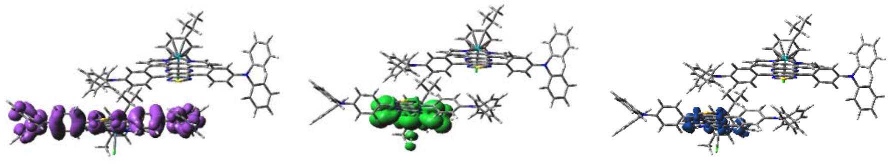

S$_{10}$
2.28 eV
f = 0.0007

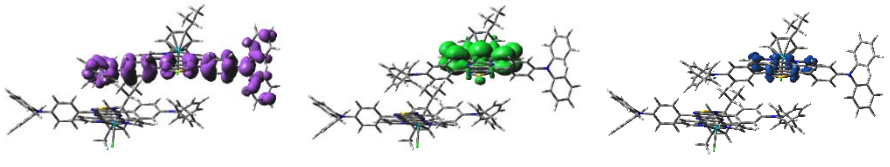

Supplementary Table 3. Contribution ratio (η) of RuDA in dimeric form for the electron transition from one RuDA molecule to the other one for the low-energy singlet excited states.

	S_1	S_2	S_3	S_4	S5	S_6	S_7	S_8	S_9	S_{10}
η (%)	2.5	0.6	95.6	93.4	0.1	0	99.8	99.7	0.2	0.3

Supplementary Table 4. Singlet and triplet excited states transition configurations of monomeric RuDA revealed by TD-DFT calculations.

	n	Energy (eV)	Transition configuration
S_n	1	1.2987	H → L (100%)
	2	1.6352	H-1→ L (99%)
	3	2.2761	H → L+1 (99%)
	4	2.3938	H-2 → L (98%)
	5	2.6052	H-1→ L+1 (61%), H → L+2 (38%)
	6	2.6655	H-1 → L+1 (39%), H → L+2 (59%)
	7	2.7410	H-3 → L (86%), H-3 → L+1 (7%), H-3 → L+4 (5%)
	8	2.7608	H → L+3 (80%), H → L+4 (15%)
	9	2.7813	H-17 → L (18%), H-9 → L (23%), H-5 → L (54%)
	10	2.8287	H-3 → L (11%), H-3 → L+1 (13%), H-3 → L+3 (18%), H-3 → L+4 (41%), H-4 → L+5 (7%)
	11	2.9302	H-4 → L (29%), H-4 → L+1 (13%), H-4 → L+4 (35%), H-4 → L+3 (9%)
	12	2.9821	H-4 → L (66%), H-4 → L+3 (10%), H-4 → L+4 (15%)
	13	2.9940	H-1 → L+2 (97%)
	14	3.0683	H → L+3 (15%), H → L+4 (83%)
	15	3.0861	H-11 → L (11%), H-7 → L (83%), H-10 → L (3%)
	16	3.1641	H-1→ L+3 (82%), H-1 → L+4 (12%)
	17	3.1835	H-3 → L+5 (66%) H-20 → L+5 (3%), H-12 → L+4 (5%), H-4 → L+1 (2%), H-3 → L+2 (7%), H-3 → L+8 (2%)
	18	3.2308	H-17 → L (40%), H-5 → L (38%) H-16 → L (5%), H-15 → L (2%), H-9 → L (8%), H-8 → L (2%)
	19	3.2446	H-10 → L (12%), H-6 → L (81%), H-11 → L (2%)
	20	3.2845	H-3 → L+1 (74%), H-3 → L+4 (13%), H-4 → L+5 (4%), H-2 → L+1 (3%)
T_n	1	0.6494	H-2 → L (24%), H → L (75%)
	2	1.4309	H-1 → L (98%)
	3	1.7199	H-2 → L (65%), H → L (26%), H-16 → L (3%)
	4	2.2025	H-3 → L+1 (10%), H-3 → L+3 (19%), H-3→ L+4 (57%), H-20 → L+4 (3%), H-4 → L+5 (2%)
	5	2.2430	H → L+1 (91%), H → L+3 (3%)
	6	2.3714	H-17 → L (21%), H-9 → L (15%), H-5 → L (21%), H → L+3 (16%), H-2 → L+3 (5%), H → L+1 (4%), H → L+4 (4%)
	7	2.4130	H-4 → L+3 (18%), H-4 → L+4 (51%), H-19 → L+4 (2%), H-4 → L+1 (9%), H-4 → L+7 (2%), H-3 → L+5 (4%)
	8	2.4669	H → L+2 (67%), H-15 → L (2%), H-4 → L (3%), H-2 → L+2 (5%), H-1 → L+1 (4%), H → L+3 (3%)

	9	2.4728	H-5 → L (11%), H → L+3 (29%), H-18 → L (3%), H-17 → L (7%), H-16 → L (3%), H-9 → L (7%), H-2 → L+3 (7%), H → L+2 (6%), H → L+4 (8%)
	10	2.5575	H-3 → L+5 (73%), H-20 → L+5 (5%), H-4 → L+4 (3%), H-3 → L+2 (5%), H-3 → L+8 (3%)
	11	2.6158	H-1 → L+1 (87%) H-15 → L (5%), H-4 → L (3%)
	12	2.6861	H-15 → L (35%), H-4 → L (26%), H >L+2 (12%)
	13	2.7264	H-3 → L (83%), H-3 → L+1 (5%)
	14	2.8013	H-4 → L+5 (62%), H-15 → L+5 (4%), H-12 → L+5 (3%), H-4 → L+2 (6%), H-4 → L+8 (3%), H-3 → L (4%)
	15	2.8416	H-1 → L+2 (20%), H → L+6 (23%), H-18 → L (4%), H-18 → L+1 (2%), H-4 → L+5 (5%), H-3 → L (5%), H-1 → L+9 (3%), H → L+3 (4%)
	16	2.8694	H-12 → L+4 (23%), H-12 → L+5 (11%), H-19 → L+3 (2%), H-19 → L+4 (7%), H-19 → L+5 (7%), H-15 → L+3 (2%), H-15 → L+4 (7%), H-15 → L+5 (2%), H-12 → L+1 (3%), H-12 → L+3 (8%), H-12 → L+7 (2%), H-4 → L+5 (4%)
	17	2.9216	H-16 → L (10%), H → L+6 (25%), H-18 → L (3%), H-18 → L+1 (7%), H-16 → L+1 (2%), H-3 → LUMO (5%), H-3 → L+1 (7%), H → L+3 (4%)
	18	2.9496	H-19 → L+5 (13%), H-12 → L+4 (10%), H-12 → L+5 (30%) H-19 → L+4 (3%), H-15 → L+4 (3%), H-15 → L+5 (8%), H-12 → L+1 (2%), H-12 → L+3 (3%), H-12 → L+8 (2%)
	19	2.9705	H-4 → L (12%), H-1 → L+3 (26%), H-1 → L+6 (10%), H-11 → L (3%), H-7 → L (9%), H-1 → L+4 (6%), H → L+9 (7%), H → L+15 (5%)
	20	2.9915	H-11 → L (11%), H-7 → L (60%), H-10 → L (5%), H-4 → L (9%), H-1 → L+2 (3%)

Supplementary Table 5. Singlet and triplet excited states transition configurations of dimeric RuDA revealed by TD-DFT calculations.

	n	Energy (eV)	Transition configuration
S_n	1	1.2702	H-1 → L (71%), H → L (3%), H → L+1 (24%)
	2	1.2829	H-1 → L (24%), H → L (22%), H → L+1 (51%)
	3	1.2939	H-2 → L (12%), H → L (62%), H → L+1 (23%)
	4	1.3053	H-3 → L+1 (14%), H-1 → L+1 (83%), H-3 → L (2%)
	5	1.5898	H-3 → L (32%), H-2 → L (10%), H-2 → L+1 (53%), H-3 → L+1 (5%)
	6	1.6071	H-3 → L (58%), H-2 → L+1 (34%), H-3 → L+1 (4%), H-2 → L (3%)
	7	1.6892	H-2 → L (73%), H-2 → L+1 (11%), H → L (12%)
	8	1.7034	H-3 → L+1 (75%), H-1 → L+1 (16%), H-3 → L (7%)
	9	2.2714	H → L+3 (99%)
	10	2.2784	H-1 → L+2 (98%)
	11	2.3360	H-3 → L+3 (12%), H-1 → L+3 (88%)
	12	2.3567	H-5 → L (31%), H-4 → L (50%), H-4 → L+1 (16%)
	13	2.3693	H-5 → L+1 (61%), H-4 → L (17%), H-4 → L+1 (18%)
	14	2.4161	H → L+2 (96%) H-2 → L+2 (3%)
	15	2.5120	H-5 → L+1 (27%), H-4 → L+1 (62%), H-5 → L(4%), H-4 → L (6%)
	16	2.5175	H-5 → L (61%), H-5 → L+1 (10%), H-4 → L (25%), H-4 → L+1 (3%)
	17	2.5814	H-2 → L+3 (73%), H → L+5 (24%)
	18	2.5935	H-3 → L+2 (69%), H-1 → L+4 (29%)
	19	2.6492	H-2 → L+3 (25%), H → L+5 (67%), H → L+4 (3%)
	20	2.6563	H-3 → L+2 (29%), H-1 → L+4 (64%)
T_n	1	0.6479	H-4 → L (18%), H-1 → L (66%), H-5 → L (9%), H-4 → L+1 (3%), H-1 → L+1 (8%)
	2	0.6480	H-5 → L+1 (19%), H → L+1 (67%), H-4 → L+1 (7%), H → L (8%)
	3	1.2930	H-2 → L (13%), H → L (76%), H → L+1 (9%)
	4	1.3022	H-3 → L+1 (16%), H-1 → L+1 (73%), H-1 → L (9%)
	5	1.3992	H-2 → L (11%), H-2 → L+1 (87%)
	6	1.4267	H-3 → L (87%), H-3 → L+1 (10%)
	7	1.6798	H-5 → L (11%), H-4 → L (35%), H-2 → L (12%), H-1 → L (21%), H-4 → L+1 (4%), H → L (3%)
	8	1.6830	H-5 → L+1 (40%), H-4 → L+1 (16%), H → L+1 (24%), H-5 → L (5%), H → L (3%)
	9	1.6909	H-2 → L (62%), H-5 → L (5%), H-4 → L (5%), H-2 → L+1 (8%), H-1 → L (5%), H → L (9%)
	10	1.7031	H-3 → L+1 (71%), H-1 → L+1 (16%), H-3 → L (8%)
	11	2.1915	H-7 → L+2 (10%), H-7 → L+6 (15%), H-7 → L+8 (52%), H-41 → L+8 (2%), H-7 → L+9 (9%)

	12	2.1971	H-6 → L+3 (10%), H-6 → L+7 (15%), H-6 → L+9 (52%), H-40 → L+9 (2%), H-6 → L+8 (9%)
	13	2.2409	H → L+3 (92%), H → L+7 (2%)
	14	2.2484	H-1 → L+2 (92%), H-1 → L+6 (2%)
	15	2.3356	H-3 → L+3 (12%), H-1 → L+3 (88%)
	16	2.3669	H-34 → L+1 (20%), H-19 → L+1 (10%), H-10 → L+1 (13%), H → L+7 (15%), H-34 → L (2%), H-18 → L+1 (3%), H-11 → L+1 (4%), H-5 → L+7 (4%), H → L+3 (3%), H → L+9 (3%)
	17	2.4025	H-35 → L (26%), H-18 → L (14%), H-35 → L+1 (3%), H-35 → L+6 (2%), H-19 → L (5%), H-15 → L (3%), H-14 → L (5%), H-12 → L (4%), H-11→ L (5%), H-10 → L (3%), H-1 → L+4 (4%)
	18	2.4135	H-8 → L+7 (13%), H-8 → L+9 (44%), H-38 → L+9 (2%), H-9 → L+9 (3%), H-8 → L+3 (8%), H-8 → L+8 (8%), H-6 → L+11 (5%)
	19	2.4142	H-9 → L+6 (12%), H-9 → L+8 (43%), H-9 → L+2 (8%), H-9 → L+9 (8%), H-8 → L+8 (3%), H-7 → L+10 (5%)
	20	2.4161	H → L+2 (96%), H-2 → L+2 (3%)
