# Supplementary material for: A supramolecular photosensitizer derived from an Arene-Ru(II) complex self-assembly for NIR activated photodynamic and photothermal therapy
Source: Nat Commun. 2022 Jun 2;13:3064. doi: 10.1038/s41467-022-30721-w (PMC9163081; doi:10.1038/s41467-022-30721-w)
Supplement: Supplementary file 3 — Reporting Summary [file 41467_2022_30721_MOESM3_ESM.pdf]

nature portfolio

Corresponding author(s): Jian Zhao, Xing-Hua Xia & Shaohua Gou

Last updated by author(s): Apr 26, 2022

Reporting Summary

Nature Portfolio wishes to improve the reproducibility of the work that we publish. This form provides structure for consistency and transparency in reporting. For further information on Nature Portfolio policies, see our Editorial Policies and the Editorial Policy Checklist.

Statistics

For all statistical analyses, confirm that the following items are present in the figure legend, table legend, main text, or Methods section.

n/a	Confirmed	
☐	☒	The exact sample size (*n*) for each experimental group/condition, given as a discrete number and unit of measurement
☐	☒	A statement on whether measurements were taken from distinct samples or whether the same sample was measured repeatedly
☐	☒	The statistical test(s) used AND whether they are one- or two-sided<br>*Only common tests should be described solely by name; describe more complex techniques in the Methods section.*
☐	☒	A description of all covariates tested
☒	☐	A description of any assumptions or corrections, such as tests of normality and adjustment for multiple comparisons
☐	☒	A full description of the statistical parameters including central tendency (e.g. means) or other basic estimates (e.g. regression coefficient) AND variation (e.g. standard deviation) or associated estimates of uncertainty (e.g. confidence intervals)
☐	☒	For null hypothesis testing, the test statistic (e.g. *F*, *t*, *r*) with confidence intervals, effect sizes, degrees of freedom and *P* value noted<br>*Give P values as exact values whenever suitable.*
☒	☐	For Bayesian analysis, information on the choice of priors and Markov chain Monte Carlo settings
☒	☐	For hierarchical and complex designs, identification of the appropriate level for tests and full reporting of outcomes
☒	☐	Estimates of effect sizes (e.g. Cohen's *d*, Pearson's *r*), indicating how they were calculated

Our web collection on statistics for biologists contains articles on many of the points above.

Software and code

Policy information about availability of computer code

Data collection	DFT and TD-DFT calculations were carried out at the PBE1PBE/6-31G*//LanL2DZ level using the Gaussian 16 program. BD FACSDiva and Cell Quest Pro softwares were used for flow cytometry data collection. The UV/Vis Spectra data were collected on a UV Probe 2.51 program. HPLC data were acquired by Empower 3 software. GPC data were collected on a Chromeleon 7.2.5.0 program.
Data analysis	Data analysis for flow cytometry was performed using FlowJo (10.8.1, USA). All statistical analyses were performed on Origin 2018 (64 bit), Excel 2016, and GraphPad Prism 8.0.1.

For manuscripts utilizing custom algorithms or software that are central to the research but not yet described in published literature, software must be made available to editors and reviewers. We strongly encourage code deposition in a community repository (e.g. GitHub). See the Nature Portfolio guidelines for submitting code & software for further information.

Data

Policy information about availability of data

All manuscripts must include a data availability statement. This statement should provide the following information, where applicable:

- Accession codes, unique identifiers, or web links for publicly available datasets
- A description of any restrictions on data availability
- For clinical datasets or third party data, please ensure that the statement adheres to our policy

All data supporting the findings of this study are available within the paper and its Supplementary Information files. The related source data are provided.

March 2021

Field-specific reporting

Please select the one below that is the best fit for your research. If you are not sure, read the appropriate sections before making your selection.

☒ Life sciences ☐ Behavioural & social sciences ☐ Ecological, evolutionary & environmental sciences

For a reference copy of the document with all sections, see nature.com/documents/nr-reporting-summary-flat.pdf

Life sciences study design

All studies must disclose on these points even when the disclosure is negative.

Sample size	Sample size were determined according to previous experimental experiences and/or well established reported protocols (Adv. Funct. Mater. 2021, 31, 2008325; J. Med. Chem. 2021, 64, 12877; Chem. Sci., 2021, 12, 11810). No statistical methods were used to predetermine sample sizes. The in vivo sample size was three animals per treatment group. Three biological replicates were considered for most the in vitro studies.
Data exclusions	No data was excluded.
Replication	Experiments were repeated and the results were reproducible. Details of experimental replicates are given in the figure legends or methods.
Randomization	The mice were randomly divided into different groups for in vivo experiment. Cell samples were randomly allocated into multi-well plates.
Blinding	Blinding was not relevant to this work, because appropriate controls were included and the reported outcomes were based on non-subjective measurements.

Reporting for specific materials, systems and methods

We require information from authors about some types of materials, experimental systems and methods used in many studies. Here, indicate whether each material, system or method listed is relevant to your study. If you are not sure if a list item applies to your research, read the appropriate section before selecting a response.

Materials & experimental systems

n/a	Involved in the study	
☐	☒	Antibodies
☐	☒	Eukaryotic cell lines
☒	☐	Palaeontology and archaeology
☐	☒	Animals and other organisms
☒	☐	Human research participants
☒	☐	Clinical data
☒	☐	Dual use research of concern

Methods

n/a	Involved in the study	
☒	☐	ChIP-seq
☐	☒	Flow cytometry
☒	☐	MRI-based neuroimaging

Antibodies

Antibodies used	The primary antibodies including Rabbit anti-β-actin (ABCAM ab68226), anti-NRF2 (ABCAM ab62352), anti-HO-1 (ABCAM ab68477), and anti-HSP70 (ABCAM ab181606) were purchased from Abcam. The secondary antibodies (Goat Anti-rabbit IgG, IgG-HRP KGAA35) were obtained from KeyGEN Biotec Co.
Validation	All antibodies used in this study were commercially available and validated by the respective manufacturer for their use in Western Blotting.

Eukaryotic cell lines

Policy information about cell lines

Cell line source(s)	Human breast cancer cell line MDA-MB-231 was purchased from KeyGEN Biotec Co., LTD (catalog number KG033).
Authentication	No cell authentication method was used.
Mycoplasma contamination	No mycoplasma contamination was observed.
Commonly misidentified lines (See ICLAC register)	No commonly misidentified cell lines were used in this study.

Animals and other organisms

Policy information about studies involving animals; ARRIVE guidelines recommended for reporting animal research

Laboratory animals	BALB/c nude mice (female, 4-6 weeks, 16-18 g) were used as animal model in this work. The animals were hosted in an equipped animal facility with temperature at 20–26 °C (daily temperature differences ≤ 4 °C) and humidity at 40%–70%, under the same dark/light cycle (12h :12h).
Wild animals	No wild animals were used in this study.
Field-collected samples	This work did not involve field-collected samples.
Ethics oversight	The experimental protocols were approved by the Animal Care & Welfare Committee of Southeast University (Nanjing, China, permit no. SYXK20160014) and KeyGEN BioTECH Co. Ltd. (Nanjing, China, permit no. SYXK-20170040)

Note that full information on the approval of the study protocol must also be provided in the manuscript.

Flow Cytometry

Plots

Confirm that:

☒ The axis labels state the marker and fluorochrome used (e.g. CD4-FITC).

☒ The axis scales are clearly visible. Include numbers along axes only for bottom left plot of group (a 'group' is an analysis of identical markers).

☒ All plots are contour plots with outliers or pseudocolor plots.

☒ A numerical value for number of cells or percentage (with statistics) is provided.

Methodology

Sample preparation	MDA-MB-231 cells were plated into 6-well culture plates (2 mL/well) and cultured in 5% CO2 at 37 °C overnight. RuDA-NPs or RuDA were added, which were diluted to a concentration of 50 µM, and irradiated with 808 nm laser (0.5 W cm-2) for 10 min or not. After 6 h, the cells were digested with trypsin and washed twice with cold PBS. Then, cells were collected by centrifugation (2000 rpm, 5 min). The apoptosis was determined by flow cytometry using an Annexin V-FITC/PI assay kit (KeyGEN BioTECH, China) according to the manufacturer's protocol. The detailed operation as follows: cells were stained with 5µL Annexin V-FITC for 5 min in Annexin-binding buffer (10 mM HEPES, 140 mM NaCl, 2.5 mM CaCl2, pH 7.4). PI (propidium iodide) was added to cells with 5µL before incubated at room temperature for 15 min. Fluorescence of cells was measured by flow cytometer (FAC Scan, Becton Dickenson, USA).
Instrument	BD FACSCalibur flow cytometer was used for data acquisition.
Software	BD FACSDiva and Cell Quest Pro softwares were used for data collection and FlowJo V10 was used for data analysis.
Cell population abundance	At least 10,000 cell events were recorded from each sample.
Gating strategy	Forward scatter (FSC) and side scatter (SSC) were used to find viable, single cells.

☒ Tick this box to confirm that a figure exemplifying the gating strategy is provided in the Supplementary Information.
